# Ventricular fibrillation arrest in aortic dissection presenting as myocardial injury

**DOI:** 10.1016/j.radcr.2024.08.140

**Published:** 2024-09-19

**Authors:** Som Singh, Talal Asif

**Affiliations:** a University of Missouri Kansas City, School of Medicine, Kansas City, MO, USA; b University of Texas, Health Sciences Center at Houston, Houston, TX, USA

**Keywords:** Aortic dissection, Myocardial ischemia, Acute coronary syndrome, Ventricular fibrillation

## Abstract

An acute aortic dissection can be a tremendously fatal vascular condition if not managed promptly. However, the symptom profile of aortic dissections can be ambiguous to numerous conditions which are more common and greater pursued by clinicians before suspicion of dissection is made. The case presented in this study is of a 61-year-old male who arrives to the emergency department for concern of new-onset chest pain which progresses into ventricular fibrillation arrest prior to diagnosis of aortic dissection. This case report profiles the mimicking possibility of aortic dissection to present as acute myocardial injury, and the utility of models use to differentiate the workup between aortic dissections and acute coronary syndromes.

## Introduction

The aorta is the largest artery of the body, and a tear through the intimal layer is lethal when all layers are involved in a full dissection [1]. Let alone, the mortality of aortic dissection is approximately 1 in every 3 individuals if left unmanaged within the first 24 hours of onset and can be nearly 50% within the first 48 to 72 hours since onset [2,3]. While this high mortality rate has led to imperative attention towards prompt management of the condition, it remains difficult to diagnose in the emergency department setting due to ambiguity of symptom presentation [4]. Moreover, symptoms of chest pain presentation can mimic conditions such as myocardial injury and may delay in diagnosing the aortic dissection. The case presented in this study demonstrates the clinical decision making in a patient presenting with acute chest pain and subsequent ventricular fibrillation arrest which mimicked acute coronary syndrome in the setting of an aortic dissection.

## Case

**Fig. 1:**
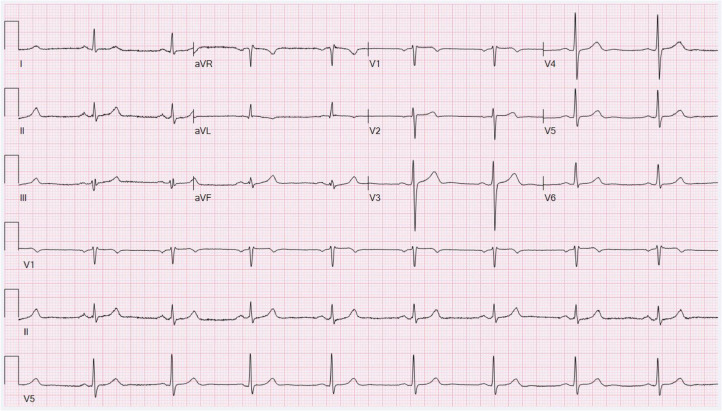
Initial electrocardiogram of patient performed immediately on onset of chest pain demonstrating sinus rythym.

A 61-year-old male patient with a medical history of hypertension, hyperlipidemia, and alcohol use disorder presented to the emergency department due to concern of new-onset, diffuse, chest, and abdominal pain. This pain started at work in the morning, and he denied any possible triggers. In the emergency department, he was found to be hypertensive at 185mmHG/54mmHg with a heart rate of 79 beats per minute. His vitals were otherwise stable with respirations of 18 breaths per minute, arterial oxygen saturation of 96% on room air, and temperature of 97.9°F. High-sensitivity troponins were elevated by approximately 3500 pg/mL and the initial electrocardiogram demonstrated sinus rhythm with regular rate (Fig. 1).

**Fig. 2:**
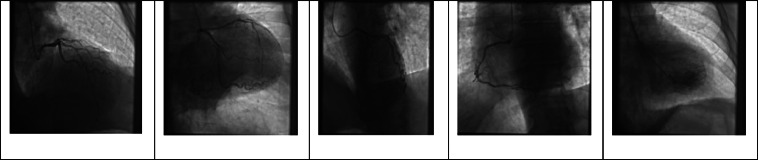
Cardiac catheterization demonstrating regular flow across right and left coronary arteries.

**Fig. 3:**
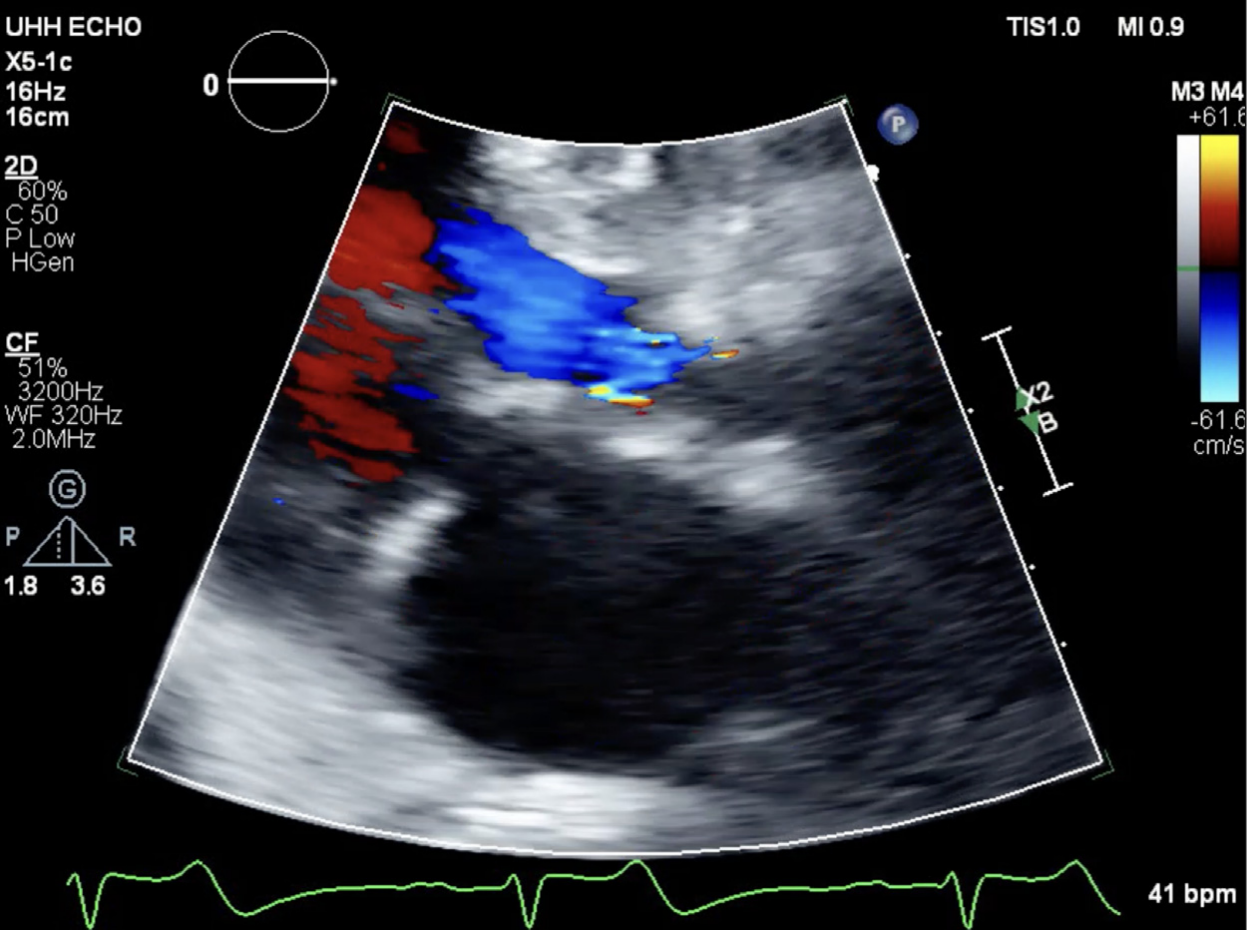
Cardiac transthoracic echocardiography demonstrating severe aortic regurgitation.

**Fig. 4:**
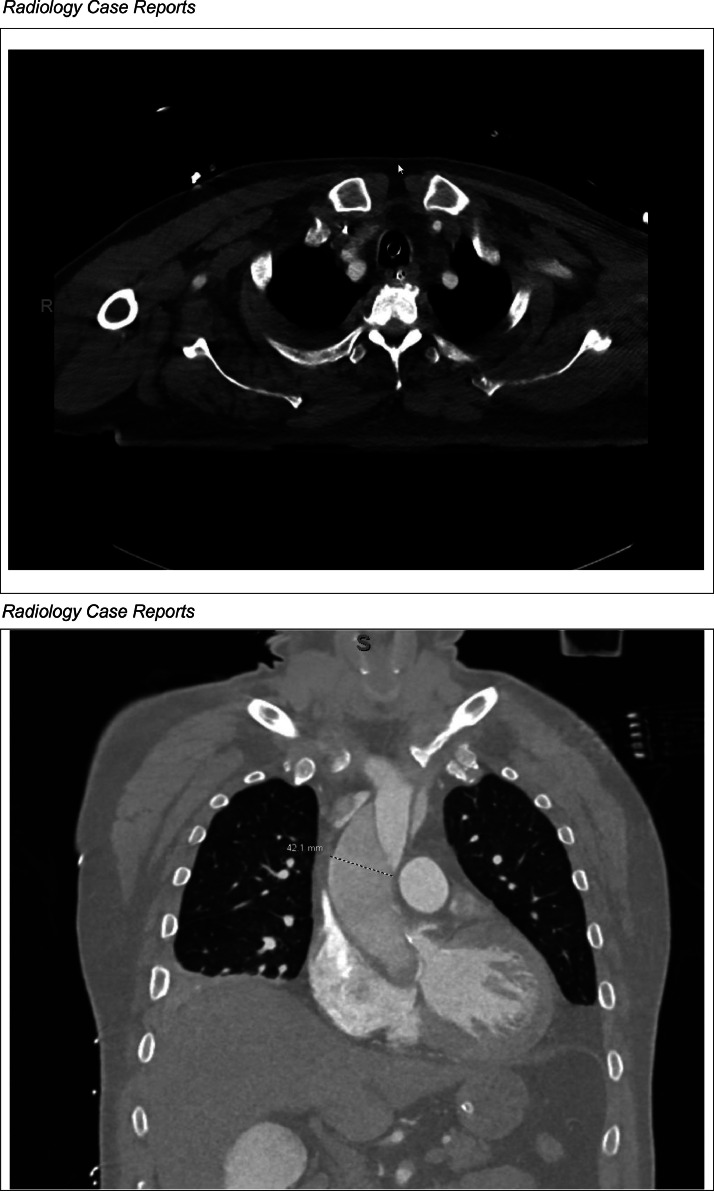
Computed tomographic angiography demonstrating 42.1 mm diameter of the mid-ascending aorta with dissection flaps.

Approximately 4 hours into this patient's emergency department course, this patient undergoes cardiac arrest with ventricular fibrillation. He is immediately defibrillated with 1 shock of 120 joules leading to return of spontanous circulation. To evaluate for acute coronary syndrome, the patient underwent left heart catheterization via radial access and demonstrated nonobstructive coronary vessels, and an intraaortic balloon pump was placed due to severely elevated left ventricle end-diastolic pressure (Fig. 2). Approximately 7 hours into his hospital course, he experienced a second episode cardiac arrest with pulseless electrical activity arrest in the setting of hypoxia that degenerated into ventricular fibrillation. He was subsequently intubated and required on vasopressor support and veno-arterial extracorporeal membrane oxygenation (VA-ECMO) for the management of cardiogenic shock. In the intensive care unit, a transthoracic echocardiogram demonstrated inferior wall hypokinesia with severe aortic regurgitation and dilated ascending aorta prompting concern extra-cardiac causes of shock including possible dissection (Fig. 3). A subsequent chest computed tomography angiography showed an increased diameter of the mid-ascending aorta at 42.1 mm and dissection flaps in the ascending and descending aorta (Fig. 4). Approximately 11 hours into his hospital course. He subsequently underwent ascending aneurysm repair with a 34 mm Hemashield Platinum woven vascular graft, and repair of the descending aorta with placement of a 37 mm Gore TAG® stent.

## Discussion

Aortic dissection is a rare but tremendously fatal disease if not treated appropriate and timely manner. Let alone, aortic dissections are found to occur approximately 5 per 100,000 visits to the emergency department annually [5]. The case presented in this study demonstrated acute abdominal aortic dissection with the initial presentation of sudden onset chest pain. With this presentation, the concern for myocardial ischemia is often reflexive among emergency departments worldwide as vascular disruption of the myocardium is one of the most common causes of mortality worldwide and troponins are subsequently measured for further diagnostic confirmation [[6], [7], [8], [9], [10], [11]]. This makes early diagnosis of aortic dissection challenging from subjective history alone given the overlapping symptom profile shared by acute coronary syndrome as well as other alternative noncardiac conditions including pulmonary embolism, costochondritis, etc. Moreover, the current body of literature suggests that missing the initial diagnosis of aortic dissection can range between 1 in every 3 patients, citing patient comorbidities such as prior cardiac disease and the lack of imaging findings on initial chest X-ray workup such as a widened mediastinum [3,12].

In addition to the imperative timing to diagnosis of aortic dissection, the symptom overlap with acute coronary syndrome may lead to clinicians to administer thrombolytic and anticoagulants per diagnostic algorithms, which can be fatal for patients with this aortic disease [13]. Moreover, a review of literature in 2015 demonstrated 10 out of 15 patients underwent percutaneous coronary intervention in the setting of type A aortic dissection that was worked up for acute myocardial infarction [14]. It would be very possible this patient could be recommended to start anticoagulation in the setting of no obstruction in this patient coronary vessels found on diagnostic angiography. However, there immediate deterioration of the patient into cardiac arrest accelerated diagnosis of the dissection with CT imaging. Most literature indicates CT imaging of the chest and aorta to confirm dissections although other modalities including transthoracic or transesophageal echocardiography, and magnetic resonance imaging could also be implemented in theory [3,15]. However, standard utilization of CT imaging may not be technical feasible across most hospitals and may be cumbersome in utility given the given the variability in symptom presentation across patients [16]. As a result, there is a growing body of quality improvement literature to develop predictive nomograms and models that can aid in the clinical decision making for opting to workup for a patient presenting to the emergency room, with 1 model predicting the probability of aortic dissection at 0.919 [17,18]. However, the variables used in this model may be ambiguous for the case presented in this study. Specifically, variables of pain severity and presences of tearing pain were utilized and this patient's lack of description for tearing pain and new onset chest pain makes the use of a nomogram model have dubiety. Moreover, this case presentation further highlights the growing importance of utilizing noninvasive imaging modalities in chest pain management. In the acute setting, chest x-ray may be able to provide rapid, efficent diagnostic rule out of other causes of this pain, such as osesophageal rupture or pnuemothorax. Additionally, the workup of myocardial injury or non-ST segment elevation myocardial infarction may benefit from further imaging modalities including cardiac CT or magnetic resonance imaging (MRI) to evaluate cardiac structure and function. Further ischemic workup may be performed through nuclear perfusion imaging as well [19].

This patient experienced ventricular fibrillation cardiac arrest twice during this hospital course. Initially, the first episode of arrest prompted this patient to undergo cardiac catheterization despite unremarkable ECG findings. This further narrowed the initial diagnosis in acute coronary syndrome over other causes of new-onset chest pain. This tendency to efficiently rule out acute coronary syndrome has been a longstanding practice in the emergency department setting [20]. The second episode of arrest after catheterization in addition to subsequent aortic regurgitation on echocardiography demonstrated concern for possible dissection that prompted immediate CT angiography. Moreover, the presence of severe aortic regurgitation on transthoracic echocardiography has been shown in previous literature where patients had an initial findings of non-ST segment elevation and aortic regurgitation found to have aortic dissection [[21], [22], [23], [24]]. Another possible solution suggested in the literature may be through standardized triple rule-out CTA to broaden diagnostic findings compared to the former [[25], [26], [27]]. However, this solution also requires further investigation on the implementation of this in the emergency department setting.

## Conclusion

Diagnosis of aortic dissection requires prompt distinguishing from acute myocardial injury workup in the emergency department setting. This case highlights the eventual diagnosis of dissection following ventricular fibrillation arrest which was initially worked up through cardiac catheterization. CT imaging can confirm diagnosis prior to management of the condition, and there are developing clinical judgement models to help navigate dissection presentations. Future investigation on these models will enable clinicians to efficiently care for the patients.

## Patient consent

Consent was obtained for publishing this de-identified case.

## Appendix Supplementary materials

Video 1: Parasternal long axis image showing a dissection flap at the level of sinuses of Valsalva and aortic regurgitation across the aortic valve. Image, video 1

Video 2: Video showing scrolling through axial imaging data set of CT thoracic aortic dissection protocol showing extensive type A acute aortic dissection. Image, video 2
